# LGBTQ+ OLDER ADULTS AND COMMUNITY NEEDS IN METRO DETROIT: ADAPTING METHODS TO EVOLVING CONDITIONS

**DOI:** 10.1093/geroni/igac059.637

**Published:** 2022-12-20

**Authors:** Angela Perone

**Affiliations:** University of California - Berkeley, Berkeley, California, United States

## Abstract

Gerontological researchers have long called for more research on diverse older adults, including diverse communities of lesbian/gay/bisexual/trans*/queer+ (LGBTQ+) older adults (e.g., race/ethnicity, economic status, religion). This paper identifies lessons learned from a community-based participatory action research (CBPR) project with LGBTQ+ older adults across four counties. Initiated by LGBTQ+ older adults of color and transgender older adults concerned about access to affordable and culturally responsive housing as they age, researchers worked with a coalition of 8 LGBTQ+ focused organizations to design, implement, and analyze a mostly online survey (n=270). Topics include: Capturing diverse perspectives in survey design (8 focus groups, N=35); addressing external threats to data integrity; implementing multiple types of outreach in phases to expand participant diversity; and working with the coalition and other partners through different stages of survey design, recruitment, data analysis, and identification/enactment of goals for change informed by data, while also navigating a pandemic.

